# Hydrodynamics of fossil fishes

**DOI:** 10.1098/rspb.2014.0703

**Published:** 2014-08-07

**Authors:** Thomas Fletcher, John Altringham, Jeffrey Peakall, Paul Wignall, Robert Dorrell

**Affiliations:** 1 School of Earth and Environment, University of Leeds, Leeds, West Yorkshire LS2 9JT, UK; 2 School of Biology, University of Leeds, Leeds, West Yorkshire LS2 9JT, UK

**Keywords:** biomechanics, comparative anatomy, fishes, functional morphology, hydrodynamics, locomotion

## Abstract

From their earliest origins, fishes have developed a suite of adaptations for locomotion in water, which determine performance and ultimately fitness. Even without data from behaviour, soft tissue and extant relatives, it is possible to infer a wealth of palaeobiological and palaeoecological information. As in extant species, aspects of gross morphology such as streamlining, fin position and tail type are optimized even in the earliest fishes, indicating similar life strategies have been present throughout their evolutionary history. As hydrodynamical studies become more sophisticated, increasingly complex fluid movement can be modelled, including vortex formation and boundary layer control. Drag-reducing riblets ornamenting the scales of fast-moving sharks have been subjected to particularly intense research, but this has not been extended to extinct forms. Riblets are a convergent adaptation seen in many Palaeozoic fishes, and probably served a similar hydrodynamic purpose. Conversely, structures which appear to increase skin friction may act as turbulisors, reducing overall drag while serving a protective function. Here, we examine the diverse adaptions that contribute to drag reduction in modern fishes and review the few attempts to elucidate the hydrodynamics of extinct forms.

## 1. Introduction

Fish diversity exceeds that of all other vertebrate groups, with extant forms demonstrating almost every conceivable feeding and locomotory adaptation. A narrative for their early evolution has been difficult to define with the sporadic stratigraphical appearance and disappearance of quite disparate groups often lacking key transitional taxa (see [1] for an excellent review). Attempts to connect overarching functional trends in locomotion with large-scale phylogenetic, ecological or environmental patterns are therefore rare. The best documented is the shift in early fish evolution from defensive exoskeletal armour to a faster, supposedly lighter morphology [2,3], although this has not been convincingly quantified.

While there is trace fossil evidence of generic fish-like behaviour, e.g. [4], it can rarely be assigned to a taxon (although see [5,6]), and preserves only a snapshot of locomotion. Thrust is coupled with drag, and movement is a hugely important constituent of overall drag, however like environmental conditions, behaviour and musculature, this information is not available from the fossil record. Therefore, the focus of this review is on passive control of flow, governed principally by gross morphology. Fluid mechanics imposes limits on what is morphologically viable in water, so it is useful to summarize the relevant physical laws.

## 2. Hydrodynamic principles

### (a) Fluid properties

When viscous forces (those holding fluid particles together) dominate, fluid flow is laminar and particles move in parallel lines. As fluid velocity increases, inertial forces dominate and the flow becomes turbulent, characterized by irregular movements, but still with average motion in the mean direction of flow. The Reynolds number (*Re*) is an expression of the ratio of inertial and viscous forces and is influenced by the animal's size. At low *Re*, the greatest influence on drag will be surface friction, moving through a relatively viscous medium with little momentum from the propulsive forces the animal generates. In larger organisms, inertial forces are more important, and adaptations are primarily aimed at preserving laminar flow or controlling turbulent boundary layers at higher speeds through relatively inviscid fluids [7]. Consequently, fish larvae must generate thrust constantly to move forwards through relatively viscous fluid [8]. There is debate as to whether changes during ontogeny reflect optimal functionality for differing *Re* values [9], or are just an energetically expensive stage of growth before achieving a streamlined adult form [10,11]. It is the point at which the *Re* is great enough for inertial forces to take priority in the design of the organism that they are broadly classified as nektonic, rather than planktonic [12].

### (b) Boundary layer development and separation

**Figure 1.**
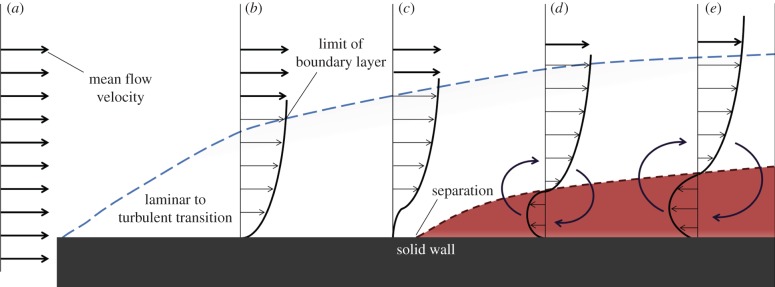
Stages of boundary layer development on a flat plate, subjected to an adverse pressure gradient. Arrows show flow direction, with length indicating velocity and mean flow velocity emboldened, boundary layer in blue and zone of vortex formation or ‘wake’ in red.

As a fluid of uniform flow (figure 1*a*) passes over a wall, molecules in contact with the surface decelerate due to shear stress from friction. The flow velocity above this decelerating fluid then becomes retarded, as particles move over slower moving particles below. Counteracting this, the fastest moving fluid in the main flow-stream above drags the underlying fluid along and a velocity profile is formed (figure 1*b*). The region between the wall to the point at which the fluid velocity is at 99% of the maximum ‘free stream’ velocity is called the boundary layer.

In an adverse pressure gradient, such as behind the widest point of a fish's body, the rising static pressure (pressure energy per unit volume) of the fluid implies a reduction of dynamic pressure (kinetic energy per unit volume) and thus a decrease in flow velocity [13] (figure 1*c*). Reduction of flow velocity induces flow to separate and reverse, forming counter rotating vortices near the wall (figure 1*d*,*e*). This is referred to as boundary layer separation, which increases the effective size of the object to be propelled through the fluid and thus also the amount of drag suffered [14].

### (c) Types of drag

Drag can be divided into three categories; pressure, induced and friction drag. Pressure drag describes the energy used to move fluid out of the way of the anterior part of the body and push it behind it again (form drag), while skin friction drag concerns the finer interactions of fluid flowing over a plane. Induced drag covers the energy lost to the component of lift force acting against the direction of motion, arising from the vortex wakes of fins and other finite lifting surfaces. Two main mechanisms of drag reduction are recognized in extant nektonic organisms; maintaining attached laminar flow as the ideal flow regime [14], or inducing and controlling turbulent flow to prevent separation [15,16].

## 3. Strategies for drag reduction in fossil fishes

### (a) Streamlining

Streamlining is a fundamental way to decrease form drag as it optimizes pressure gradients which develop across the body. Many fishes are dorsoventrally or laterally compressed (e.g. flatfishes, lookdowns, respectively), or long and torpedo-like (e.g. barracuda) to minimize their impact against the fluid as they move. Body shape should act to maintain a favourable pressure gradient and laminar flow, with the widest part of the body in the centre [16]. In some of the fastest moving fishes, protrusions from the body surface can be tucked into fairings that maintain the streamlined shape, and even the eyes do not protrude [17].

### (b) Turbulisors

**Figure 2.**
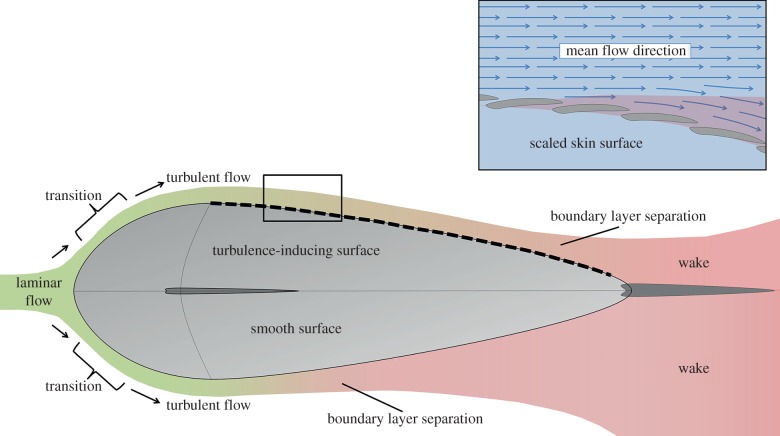
Boundary layer development and separation across a fish-like form, showing the effect of a turbulisor on flow regime and wake formation.

To delay boundary layer separation, some species (particularly fast-swimming pelagic fishes) use turbulisors at the widest point of their body to induce turbulent flow (figure 2). As water passes over the contractor region (from the anterior leading edge to the widest point of the body), laminar flow is maintained as dynamic pressure is high, pushing the fluid towards the wall. After the contractor, in the diffuser region (the narrowing area towards the tail), dynamic pressure decreases, static pressure increases and boundary layer separation may occur [12,13]. Turbulisors can include surface roughness, fins and gills, but all trigger the transition from laminar to turbulent flow which transfers some of its momentum towards the wall, meaning the boundary layer stays attached for longer. Fishes also maintain attachment by ‘blowing’ fluid from their gills (positioned at the widest point of the body) downstream, counteracting retarding flow in the boundary layer at high speeds [18].

**Figure 3.**
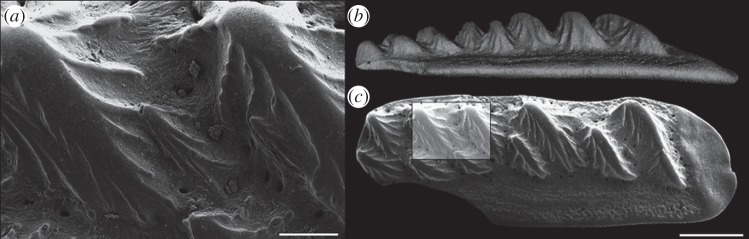
Flank scale of the osteichthyan *Lophosteus:* (*a*) scanning electron microscope (SEM) image of large buttressed tubercles on upper surface; (*b*) lateral view (surface rendering of µCt scan); and (*c*) dorsal view (SEM image). Scale bar: (*a*) 100 µm, (*b*–*c*) 0.5 mm.

**Figure 4.**
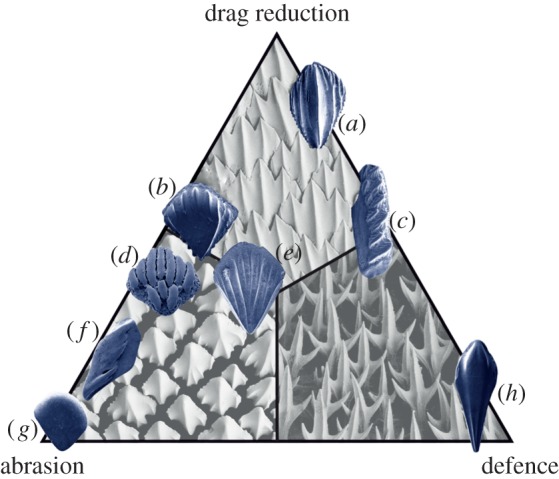
Hypothesized drag reduction, abrasion resistance and parasitic defence functions of the flank scales of (*a*) *Phlebolepis elegans*, (*b*) *Nostolepis striata*, (*c*) *Lophosteus*, (*d*) *Oniscolepis* sp., (*e*) *Thelodus laevis*, (*f*) *Andreolepis*, (*g*) *Thelodus parvidens*, and (*h*) *Loganellia cuneata*. Based on Reif's scheme of shark scale classification [19]. Background SEM images courtesy of Sue Lindsay, Australian Museum: top; *Carcharhinus obscurus*, left; *Orectolobus ornatus*, right; *Deania calcea*.

In fishes with rough scales in the diffuser region, the contractor tends to be much smoother, often composed of large bony plates, or in the case of sharks the scales are relatively smoother on the head [19]. A large number of fossil fishes have tubercles ornamenting the surface of their scales, e.g. the birkeniid anaspids *Liivilepis curvarta* and *Silmalepis erinacea* [20] and the osteichthyans *Ligulalepis toombsi* [21] and *Lophosteus* sp. (figure 3). An alternative or additional function is that these blocky backward-pointing projections could have served to protect the animal from abrasion or prevent epibiont parasite attachment, as in modern sharks (figure 4) [19].

The tubercles on the rostrum (sword) of fishes such as *Istiophorus* (sailfish) may act as a turbulisor, with the surface of the sword propagating a turbulent boundary layer which is already thick by the time it reaches the main portion of the head [12,22]. Different forms of rostral elongation are seen in a disparate array of early jawless fishes, such as galeaspids, heterostracans, osteostracans and pituriaspids, however, it is difficult to decouple feeding functions in these examples. Rostral elongation for drag reduction is more convincing in some long-snouted placoderms (e.g. *Rolfosteus*, *Carolowilhelmina* and *Oxyosteus*, e.g. [23]), which superficially resemble sailfish, but the efficacy of this adaptation in fossil fishes is untested.

### (c) Stabilizing structures and vortex control

The principle functions of the dorsal fin are to prevent roll and to enlarge the surface area giving stability during quick turns. The dorsal fin is positioned posteriorly in fishes with a pike-like (sagittiform) morphology that are capable of short bursts of rapid acceleration, with relatively little manoeuvring as they dart forwards. Fishes that require manoeuvrability during rapid and sustained swimming have their dorsal fins further forwards where they may be actively erected at critical moments and then repositioned flush to the body surface (See ‘Inferring swimming mode and ecomorphological convergence’). Alternatively, the dorsal fin acts for defence in extant species with spines (e.g. many catfishes, *Squalas acanthias*, *Heterodontus portusjacksoni*); which was presumably the function in extinct spinose species such as acanthodians and hybodont sharks.

The earliest paired fins were those of anaspids such as the Silurian *Phlebolepis*, which had long ventrolateral fins capable of undulatory propulsion [24] much like modern knifefish (Gymnotiformes). In fishes with an epicercal tail, like most sharks, the pectoral fins act to counteract the pitch of posteriorly produced lifting forces and are consequently fairly immobile. Acting in much the same way the pectoral fins of primitive Triassic teleosts are abdominal and orientated horizontally to stabilize trajectory and to a lesser degree brake. In later teleosts, the pectorals are more dorsal and hinge vertically, playing a more active role in propulsion and are sometimes the primary source of motion, e.g. *Diodon*. Pectoral fins can act as hydrofoils and produce lift, e.g. *Acipenser* and *Prionace*, (but overall importance has been questioned [25]), whereas in faster swimming fishes they are more pointed [26], acting as stabilizers. The Triassic *Potanichthys* is an exceptional case, resembling modern (and unrelated) flying fishes, its enlarged pectoral fins were probably used to glide above the water surface [27]. Pelvic fins are thought to be the least important for stabilization, which may underlie their secondary loss in several lineages including sticklebacks, true eels and seahorses (e.g. [28]).

Slow-moving fishes with negative buoyancy often have asymmetric, epicercal caudal fins, used in part to create vertical lifting forces. This may have been the case for the heavily armoured, Early Devonian pteraspid *Errivaspis waynensis* although attempts to reconstruct its hydrodynamics have focused on the underside of the bony head shield as a simple lifting surface [29], raised in pitch by the downward force of the tail. On this premise, workers have suggested that *Errivaspis* were both benthic (moving in short powered bursts [12]) and facultative pelagic planktivores [30]. Recent wind tunnel experiments have shown that the cephalic shield acts very much like a delta wing [31], creating vortices roughly parallel to the leading edge. In essence, fluid flows over these vortices and is also pulled in, accelerating as the vortex widens posteriorly, providing an important source of lift during swimming, as in modern boxfishes [32–34].

### (d) Skin friction drag

The scales of fishes have several functions including physical defence, a calcium reservoir, to prevent folding of the skin (which compromises streamlining) [12] and alteration of flow around the body. For a long time, it was assumed that achieving the smoothest possible surface was the most efficient way to reduce drag, but boundary layer separation can occur across smooth surfaces very quickly in regions of adverse pressure gradient. Additionally, even the smoothest surface produces a ‘streaky’ flow structure within the laminar sublayer, i.e. areas of low and high velocity in roughly parallel streaks. It is thought that this streaky flow directly affects the motion of vortices in the turbulent layers above [35].

Rather than having smooth skin many fast-moving sharks have placoid scales with pronounced parallel riblets which, as well as improving scale robustness, passively control flow by limiting the lateral transfer of force, training the vortices in the direction of flow [36,37]. The vortices that form are also lifted away from the wall by the riblets, reducing overall skin friction. The optimization of these riblet structures for drag reduction, in shape, spacing (typically 40–80 µm in the fastest sharks) and material, has been the focus of biomimetic applications and can achieve up to 10% reductions in skin friction [38]. Moreover, the distribution of pressure across the body surface while in motion appears to be positively influenced by the presence of placoid scales, affecting thrust as well as overall drag reduction [39]. It has been suggested that pressure fluctuations are controlled by the injection of fluid from beneath the scale, but this is yet to be tested experimentally [40]. In some of the fastest sharks, the bases of the scales are wider and shorter to accommodate pivoting, which passively forms a bristled surface to counteract regional flow reversal [41]. Interestingly, this base morphology is also found in a small number of acanthodians which also possess a ribletted crown surface.

The first riblet-like structures are found in a Middle Ordovician fish (possibly a chondrichthyan) [42], suggesting speed and efficiency were an important selection pressure even in the earliest stages of fish evolution. Riblets are not limited to chondrichthyes: it appears that within those Palaeozoic fishes that possessed scales, only a few groups lacked riblets at some point in their evolution. Placoderms are the exception [43], but their heavy exoskeleton was almost certainly primarily defensive in function. That said, the placoderm *Sedowichthys* had superficially similar structures ornamenting the dermal armour [44] (thin grooves and ridges perpendicular to the outer edge) that could have been significant for drag reduction. However, determining the physical relief of these structures in placoderms is difficult, because of the possibility of thick overlying soft tissue [45–47].

Ctenii are the small comb-like projections found on the posterior edge of ctenoid scales in teleosts and a limited number of other groups (e.g. Polyodontidae [48]). There is little discussion of their function, and suggestions that they ‘comb’ the boundary layer to control the transition to turbulent behaviour [12] have not been tested experimentally. Ctenii would actually increase turbulence if they were large enough, but are considered subroughness within the laminar sublayer, having little effect on friction drag. While their morphology may be influenced by other factors, e.g. skin flexure [49], it is likely that their presence increases the surface area from which mucus can dissolve into the fluid stream [15].

Depending on ecology, selection pressures favour different scale functions, typified by the sharks and rays whose scales have four functional extremes [19,50]; defence, abrasion resistance, luminescence (not addressed here, but see [51]) and drag reduction. Other fishes resist mechanical force with plywood-like layering of the scale material [52,53], whereas placoid scales have a blocky robust shape and widely spaced, non-parallel riblets (e.g. figure 4) [19]. Defence against epibionts is also an important selection pressure, since parasites are thought to have as long a history as their fish hosts [54,55]. The same pressures persist in modern taxa, and the convergence of scale morphologies between Palaeozoic fishes and extant sharks is remarkable.

It is important to note that some of the fastest fishes, such as tuna and billfishes, have almost completely (sometimes ontogenetically) lost their scales, and the small v-shaped scales of sailfish serve a negligible drag reduction function [56]. Some of the fastest sharks too, have evolved relatively dense, but lighter and thinner scale crowns, thought to improve scale packing [57]. The loss of dermal skeletal mass is a unifying trend throughout the evolution of many groups of fishes [58] and is well documented in the Triassic belone-like fish *Saurichthys* (figure 3*a*) [59]. However, scale loss in the context of drag reduction is poorly understood, and there are several potentially more significant factors affecting scale mass, such as calcium storage and defence.

## 4. Tail morphology and its functional significance in fossil fishes

As the primary producer of thrust, the tail is an important aspect of fish locomotion and has historically been discussed in the context of counteracting negative buoyancy (e.g. [30,60–64]). Given the movement of the tail during swimming, decoupling active and passive flow control is difficult to justify, and studies using static models are of limited value [65]. Recent studies of the hydrodynamics of the epicercal tail of modern sharks (e.g. [66–68]) are of more use, as they constrain the possible behaviours with a given morphology.

An asymmetrical (heterocercal) tail allows forward propulsion, but the greater relative flexibility of the upper (hypocercal) lobe or lower (epicercal) lobe, generates forces (downward or upward, respectively) in the vertical plane. Many early fishes had a hypocercal (e.g. myllokunmigiids, hagfishes, lampreys, euconodonts, anaspids, galeaspids and most thelodonts) or epicercal tail (e.g. pituriaspids, acanthodians, placoderms, chondrichthyes and osteichthyans) [69].

Some of the earliest examples of symmetrical tails are found in the furcacaudiform thelodonts (literally ‘fork-tailed’), and some heterostracans (e.g. *Dinaspidella* [70] and *Doryaspis* [71]). In stabilizing pitch, a lobed and asymmetrical caudal fin usually corresponds to a transversely asymmetrical body shape, with a more rounded surface on the side of the longer lobe. This is possibly because the wake created by the rounded surface is higher above the skin surface and the caudal fin must extend out of the vortex zone [12]. Much like a hydrofoil, the curved surface (for example, on the upper side of a sturgeon) can reduce flow velocity relative to the flatter side, creating a pressure differential capable of creating lifting.

The tail can also indicate the likely swimming speed of the fish, because boundary layer separation at higher speeds occurs in the middle portion of the tail. In most cases, slow-swimming fishes have rounded unlobed tails, which give the fish a larger surface area for membrane stability, but perform weakly at high cruising speeds when vortices form across the surface. The solution for faster (and sustained) movement is to discard this central portion, and indeed some of the fastest fishes (e.g. Scombridae, Xiphiidae and Istiophoridae) have very concave caudal fins with narrow lobes that avoid the vortex zone [72]. The peduncle (immediately anterior to the caudal fin) tends to be narrow in these fishes, as propulsion is generated primarily from undulations of the caudal fin. Deeply concave tails are not suited to rapid acceleration and sharp direction changes, so there exists a functional ecological spectrum [12,73]. The forked tail of the Lower Triassic coelacanth *Rebellatrix divaricarca* is assumed to represent a shift to sustained fast swimming; unique in a group that generally has large rounded tails for fast acceleration [74]. While there are studies that have sought to quantify this ecomorphological correlation in modern fishes (e.g. [75–77]) fossil taxa have not received the same treatment (but see [78]).

## 5. Inferring swimming mode and ecomorphological convergence

**Figure 5.**
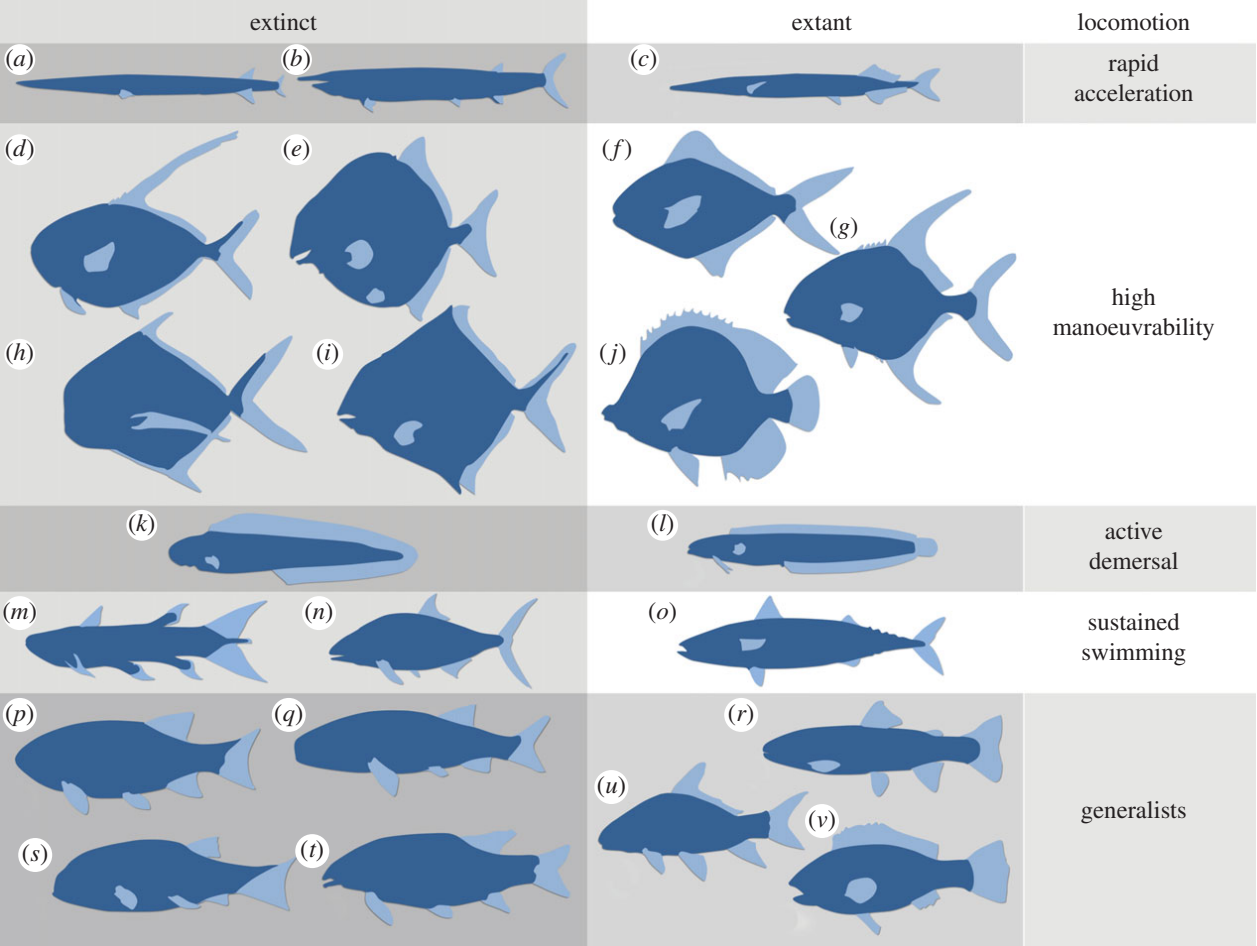
Examples of hypothesized swimming morphotypes of extinct and extant fishes: (*a*) *Saurichthys* (Triassic), (*b*) *Aspidorhynchus* (Mid-Jurassic–Late Cretaceous), (*c*) *Belone belone* (extant garfish), (*d*) *Dorypterus* (Permian), (*e*) *Proscinetes* (Jurassic), (*f*) *Stromateus fiatola* (extant pomfret), (*g*) *Trachinotus falcatus* (extant permit), (*h*) *Bobasatrania* (Triassic), (*i*) *Cheirodus* (Carboniferous), (*j*) *Chaetodon* (extant butterflyfish), (*k*) *Tarrasius* (Carboniferous), (*l*) *Clinoporus biporosus* (extant ladder klipfish), (*m*) *Rebellatrix divaricerca* (Early Triassic), (*n*) *Hypsocormus* (Mid-Late Jurassic), (*o*) *Scomber scombrus* (extant atlantic mackerel), (*p*) *Parasemionotus* (Early Triassic), (*q*) *Mesolepis* (Carboniferous), (*r*) *Oncorhynchus mykiss* (extant rainbow trout), (*s*) *Carpiodes cyprinus* (extant quillback), (*t*) *Perleidus* (Early–Middle Triassic), (*u*) *Paracentrophorus* (Early Triassic), (*v*) *Serranus hepatus* (extant brown comber). After [17,74,79,81–84].

Throughout the evolution of fishes, there have been repeated convergences on strikingly similar morphologies (e.g. [78–80] and figure 5). Both mako sharks and tuna are fast pelagic predators and have convergent external morphology, but their internal mechanical design is strikingly similar as well, despite 400 Myr of phylogenetic separation [85]. Many factors influence morphology but all relate to movement and hydrodynamics, and schemes which classify swimming morphotypes (e.g. [81]) are powerful tools for reconstructing the palaeobiology of extinct species, regardless of phylogenetic association.

### (a) Rapid acceleration

Elongate arrow-like fishes (figure 5*a*–*c*) like pike, barracuda and others, have a dorsal and anal fin positioned posteriorly, to assist the tail in bursts of rapid acceleration, but they are relatively inefficient at steady swimming [86]. The Triassic fish *Saurichthys* is superficially similar to modern garfish (*Belone belone*), which has served as an analogue for a computational fluid dynamical study, highlighting the effectiveness of this dart-like morphology [82].

### (b) High manoeuvrability

Lateral compression and deepening of the body (figure 5*d*–*j*) are often associated with high flexibility (difficult to infer in fossil taxa) in fishes such as angelfish (Pomacanthidae) and butterflyfish (Chaetodontidae) (figure 5*j*). This allows greater manoeuvrability, with a reduced ‘turning circle’ [7], as the sides of the fish offer a large surface area for braking and rapid changes in direction. Examples in the fossil record include pycnodonts [87], the acanthomorphs *Aipichthys* and *Pycnosteroides*, the osteichthyans *Ebenaqua* and *Cleithrolepis*, and the thelodont *Furcacauda*.

### (c) Active demersal

Fishes inhabiting complex demersal environments (figure 5*k–l*) tend to have elongate bodies, tapering backwards, e.g. moray eel (*Muraena helena*) and lungfishes. Such is the focus on low-speed manoeuvrability that the pectoral fins may become the primary thrust generators and become more robust to negotiate spatially challenging habitats. Conversely, species that propel themselves with anguilliform (eel-like) swimming may show a reduction or even complete loss of the pectoral fins [79].

### (d) Sustained swimming

There is always a trade-off between manoeuvrability, energetic efficiency and speed (see ‘Generalists’ figure 5*p*–*v*), and cruisers prioritize sustained high-speed swimming. These fishes (e.g. tunas and their relatives) not only have a higher aspect ratio and a more hydrodynamically optimal torpedo-like body, but also larger heads to prevent recoil energy being lost as they beat their lunate caudal fins.

### (e) Dorsoventral compression and the ground effect

Boundary layers form against all walls interacting with a flow, including riverbeds and seafloors, and there is a thin layer of lower velocity water at the interface (the laminar sublayer). By exploiting this layer, dorsoventrally compressed benthic fishes expend less energy maintaining their position at rest. Flatfishes, in particular, can withstand significant water velocities before being dislodged [7,88] and secondary migration of the eyes to accommodate this strategy can be tracked in their evolution [89].

Similar flattening is seen in the Early Devonian placodern *Gemuendina stuertzi,* the agnathan *Drepanaspis* and the thelodont *Turinia pagei,* which has been compared with the extant angelshark in form and lifestyle [90]. In addition to being flattened, some extant fishes are small enough to move in the boundary layer of fast-flowing rivers (e.g. *Etheostoma tetrazonum*), where their morphology can be surprisingly independent of hydrodynamic influences [91].

## 6. Soft tissue evidence and the limitations of fossil data

### (a) Collagen

The integument of sharks and other fishes has a highly structured mesh of collagen fibres that acts elastically to keep the skin taut and prevent folding during locomotion [92–95]. The skin can act as an external tendon, reducing the muscle contraction required to normalize shape after a power stroke. This pattern is seen in many aquatic forms, unlike terrestrial vertebrates where these fibres tend to be randomly orientated [58]. This has also been described in the aquatic mosasaurs, where the scales also possessed a keel-like ornament [96].

### (b) Mucus

The secretion of mucus can decrease surface friction in turbulent flow by up to 66% in some species and the ‘reluctance’ or relative insolubility of mucus in some species can reduce the cost of its production, since it dissolves into the water only during high-speed manoeuvres [97,98]. The only convincing evidence of a mucous coat in fossil fishes would be the preservation of an endothelial germ layer packed with goblet cells. However, modern teleost ctenoid scales are normally only found in turbulent flow regions of the body, where their comb-like spines (‘ctenii’) probably increase the surface area for mucous deposition near the wall [15]. Ctenii can be preserved in quite exceptional detail (e.g. [99]), but even if such evidence were found in Palaeozoic taxa, there is enormous variation in the drag-reducing influence of mucus from different species, and there does not appear to be a consistent correlation with swimming speed [97–101]. Mucus is clearly an important factor in fish hydrodynamics largely ignored in previous studies, however even well-informed approximations of epidermal thickness are of limited use for experimental analysis because they do not control for the volume or fluid properties of any mucus.

## 7. Future work

Despite major advances in morphometric approaches to comparative anatomy, the applications are limited, especially for unusual Palaeozoic fishes. The employment of rigorous engineering analysis methods is revolutionizing the way palaeontologists study biomechanics, and although research has focused on feeding mechanics, aquatic locomotion is now receiving attention. Modern fishes (and other marine organisms) have been a rich source of biomimetic inspiration and have helped improve our understanding of fluid mechanics. With the majority of fish species now extinct, there is a potential wealth of as yet undiscovered novel solutions to flow control in the fossil record.
